# Cerebral metastases of malignant mesothelioma

**DOI:** 10.1093/jscr/rjac002

**Published:** 2022-02-06

**Authors:** Abhijith Bathini, Dorian M Kusyk, Kristen Stabingas, Brandon Kujawski, Janice Ahn, Richard Williamson

**Affiliations:** 1 Department of Neurological Surgery, Drexel University College of Medicine, Philadelphia, PA, USA; 2 Department of Neurological Surgery, Allegheny Health Network, Pittsburgh, PA, USA

## Abstract

Malignant pleural mesothelioma represents a rare etiology of lung cancer metastasis to the brain. Neurologically symptomatic presentations are extremely rare as these metastatic lesions are detected in the late stages of the disease. Despite many highly heterogenous treatment techniques reported in the literature, overall survival is poor. A 72-year-old male with a history of mesothelioma presented with recurrent episodes of altered mental status, confusion and expressive aphasia. Imaging indicated a large hemorrhagic, enhancing lesion in the anterior left frontal lobe resulting in midline shift of 6 mm. He underwent a left frontal craniotomy for resection, after which he had complete resolution of symptoms. The resected mass was metastatic high-grade malignant mesothelioma. On a 1-month follow-up, new lesions in the bilateral frontal lobes were discovered, and despite undergoing adjuvant stereotactic radiosurgery, the right one grew significantly, causing notable mass effect. The patient successfully underwent a right craniotomy for resection.

## CASE DESCRIPTION

Malignant mesothelioma represents a growth of mesothelial cells strongly associated with asbestos exposures [1]. It can occur on any mesothelial layer such as the peritoneum or pericardium, but the pleural layer of the lungs is by far the most implicated location, thereby giving rise to the name of malignant pleural mesothelioma [1, 2]. This locally invasive tumor often spreads in the visceral pleura before extending into the chest wall, diaphragm and mediastinum. In addition, there is direct local invasion of lymph nodes, unlike other forms of lung cancer [1].

Malignant pleural mesothelioma represents a rare etiology of lung cancer metastasis to the brain with an estimated prevalence of 2–3.8% [3]. It is believed that metastatic disease enters the blood vessels and ultimately enters the central nervous system due to a disruption in the blood–brain barrier. However, these figures are determined based on post-mortem studies [4, 5]. Brain metastases are often associated with poor survival outcomes and pose unique treatment challenges as these lesions are detected in the late stages of the disease. Furthermore, there are few cases in literature, thereby making neurologically symptomatic presentation of malignant pleural mesothelioma with brain metastasis extremely rare [6, 7]. Here, we present the case of a symptomatic patient with a history of lung cancer who presented with a brain mass that was ultimately determined to be mesothelioma.

## CASE

**Figure 1:**
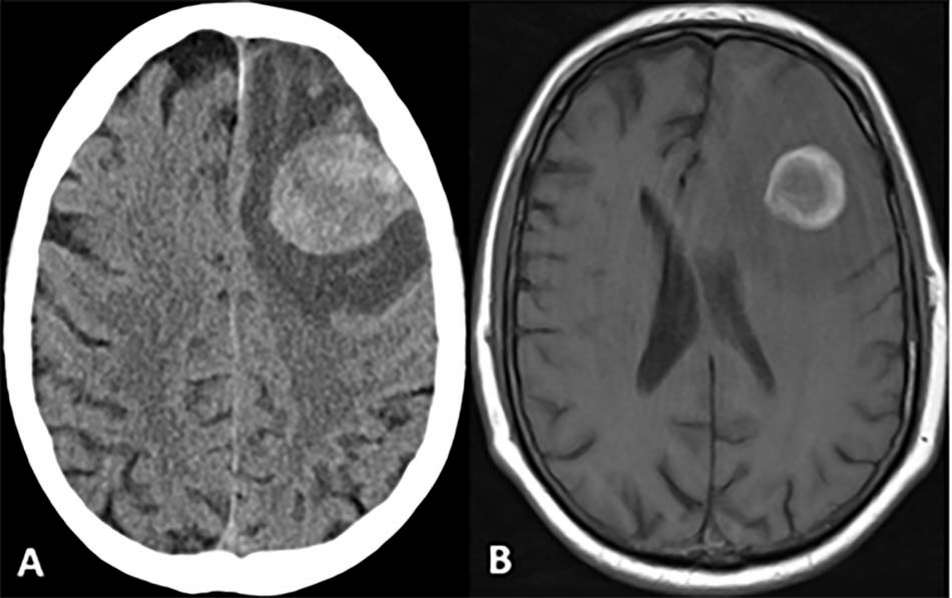
CT and MRI scans of the head indicating a large hemorrhagic enhancing lesion in the left frontal lobe with surrounding edema causing a rightward midline shift.

**Figure 2:**
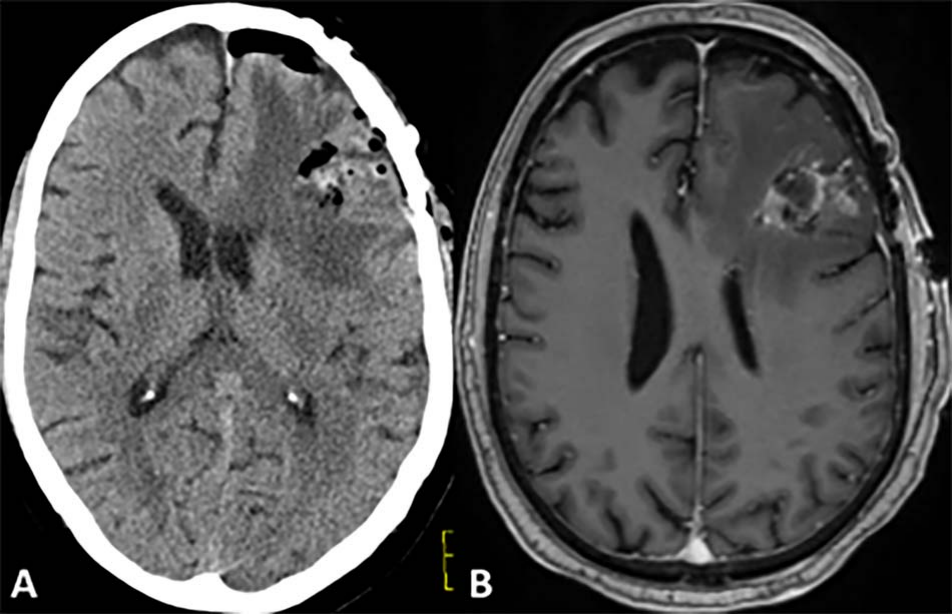
Postoperative CT and MRI scans of the head indicating complete tumor resection with reduced midline shift.

**Figure 3:**
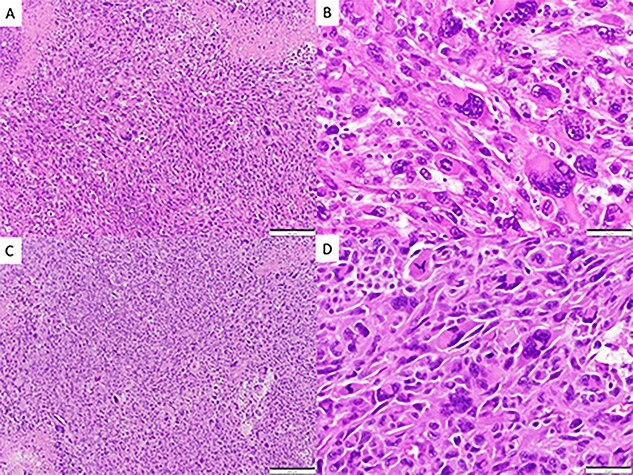
Spindle cell neoplasm with pleomorphic epithelioid features, multinucleation, mitoses and necrosis, involving pulmonary artery (**A**, **B**) and brain (**C**, **D**), H&E.

**Figure 4:**
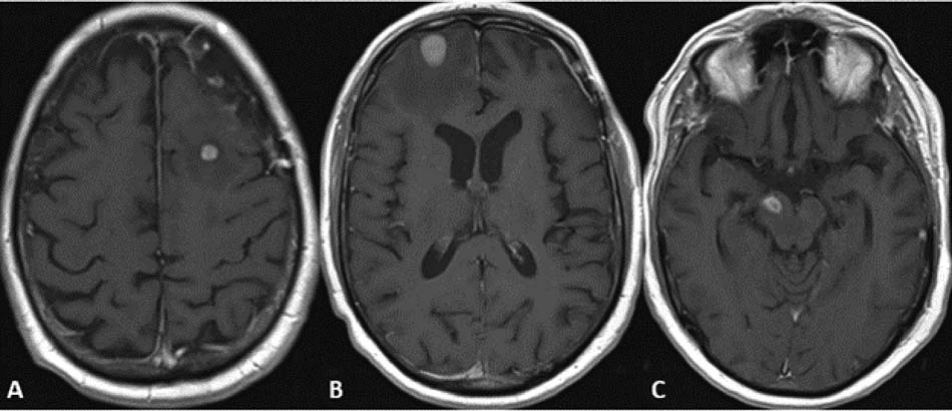
MRI scans indicating new metastatic lesions of the bilateral frontal lobes and right cerebral peduncle.

A 72-year-old male with a history of prostate adenocarcinoma for which he underwent prostatectomy a year prior and mesothelioma that was discovered during a pulmonary thromboendarterectomy 6 months prior presented to the emergency department with recurrent episodes of altered mental status, confusion and expressive aphasia of 2 days duration. According to his partner, the patient would freeze up in the middle of sentences and be unable to finish them. On physical exam, he was oriented only to person and place. Pupils were equal round and reactive. Cranial nerves were intact. He followed commands with full strength in all extremities. His sensation was intact bilaterally. He did not have any pronator drift or pathologic reflexes. A complete blood count and comprehensive metabolic panel were both within normal limits. Computed tomography (CT) and magnetic resonance imaging (MRI) scans of the head were performed which indicated a large parenchymal hemorrhagic, enhancing lesion in the anterior left frontal lobe with surrounding white matter edema resulting in a midline shift of 6 mm (Fig. 1). A secondary lesion was encountered in the right cerebellar hemisphere. The patient was started on decadron and keppra. On the following day, he underwent a left frontal craniotomy for resection of the intra-axial tumor. Postoperative CT and MRI scans indicated complete evacuation of the lesion with resolving edema and midline shift (Fig. 2). Pathology reports indicated that the resected mass was metastatic high-grade malignant mesothelioma with sarcomatoid and epithelioid features (Fig. 3). Within 5 days after the operation, the patient had complete resolution of symptoms. On a 1-month follow-up MRI scan, the prior right cerebellum lesion had grown in size and new metastatic lesions were discovered in the bilateral frontal lobes and right cerebral peduncle (Fig. 4). There was no evidence of residual or recurrent tumor in the previous resection cavity. The patient subsequently underwent adjuvant stereotactic radiosurgery (SRS). One month after SRS, a follow-up MRI scan indicated significant increase in size of the bilateral frontal lobe lesions as well as a new lesion in the right pons. The right frontal lobe lesion, in particular, was causing notable local mass effect. The remaining lesions had either slightly increased in size or were stable. Repeat CT of the abdomen and pelvis once again showed no signs of metastasis to these regions. The patient successfully underwent a craniotomy for resection of the large right frontal lesion and is scheduled to follow-up with oncology and radiation oncology for further treatment.

## DISCUSSION

The patient presented in this report had a lifelong career in the construction industry with known extensive exposure to asbestos at work sites, known to be a primary risk factor for malignant pleural mesothelioma [1]. There are three subtypes, classified based on histology: epithelioid, sarcomatoid and mixed [1, 6]. Our patient’s mixed subtype has a median survival time of 7.4–13.1 months [8]. After starting in the parietal pleura, mesothelioma most often spreads locally in the visceral pleura and onwards to the chest wall, diaphragm, mediastinum and regional lymph nodes [1, 2]. Distant metastasis is rarely seen in this form of cancer, possibly due to the rapid progression of the disease [3, 5, 6]. In this present case, the patient’s mesothelioma was first discovered 6 months prior as the patient was worked up for pulmonary embolism and persistent pulmonary hypertension. Imaging at that time showed a diffusely occlusive pulmonary vascular tumor. He subsequently underwent pulmonary thromboendarterectomy for removal of the tumor, which was found arising from the pleural surface and seated within the pulmonary artery. Further imaging indicated no signs of metastatic spread within the thoracic or abdominal cavities.

The prevalence of central nervous system metastasis from malignant mesothelioma is estimated to be ~2.7% [3]. Symptoms of metastatic spread are dependent on the involved locations. The most commonly proposed mechanism involves hematogenous spread that leads to solitary lesions in both supra- and infratentorial locations [9, 10]. Various treatment modalities such as surgical resection, immunotherapy, systemic corticosteroids, whole brain radiation, intrathecal and systemic chemotherapy have been reported. Despite these highly heterogenous treatment techniques that result in low surgical morbidity and effective symptomatic control from a neurological perspective, overall survival has been reported to be poor [3, 10].

We present a unique case in which a patient’s mesothelioma metastasized to the brain with resulting neurological deficits. As our patient was being treated with mycophenolate mofetil, had high supplemental oxygen demand and was suffering from pneumonitis, it was not feasible to treat with systemic chemotherapy. Despite being successfully treated with total gross resection, the patient had to undergo another craniotomy for resection of a new right frontal lobe lesion that was causing notable mass effect. Through this case, we aim to highlight that despite the employment of multimodal treatment approaches, including surgical excision of symptomatic lesions, further metastatic lesions can occur that complicate treatment plans.

## CONFLICT OF INTEREST STATEMENT

No authors have any conflicts financial or otherwise to declare.
